# The Relationship of COVID-19 Severity with Cardiovascular Disease and Its Traditional Risk Factors: A Systematic Review and Meta-Analysis

**DOI:** 10.5334/gh.814

**Published:** 2020-09-22

**Authors:** Kunihiro Matsushita, Ning Ding, Minghao Kou, Xiao Hu, Mengkun Chen, Yumin Gao, Yasuyuki Honda, Di Zhao, David Dowdy, Yejin Mok, Junichi Ishigami, Lawrence J. Appel

**Affiliations:** 1 Department of Epidemiology, Johns Hopkins Bloomberg School of Public Health, Baltimore, US; 2 Welch Center for Prevention, Epidemiology, and Clinical Research, US

**Keywords:** COVID-19, cardiovascular disease, risk factors, meta-analysis

## Abstract

**Background:**

Whether cardiovascular disease (CVD) and its traditional risk factors predict severe coronavirus disease 2019 (COVID-19) is uncertain, in part, because of potential confounding by age and sex.

**Methods:**

We performed a systematic review of studies that explored pre-existing CVD and its traditional risk factors as risk factors of severe COVID-19 (defined as death, acute respiratory distress syndrome, mechanical ventilation, or intensive care unit admission). We searched PubMed and Embase for papers in English with original data (≥10 cases of severe COVID-19). Using random-effects models, we pooled relative risk (RR) estimates and conducted meta-regression analyses.

**Results:**

Of the 661 publications identified in our search, 25 papers met our inclusion criteria, with 76,638 COVID-19 patients including 11,766 severe cases. Older age was consistently associated with severe COVID-19 in all eight eligible studies, with RR >~5 in >60–65 versus <50 years. Three studies showed no change in the RR of age after adjusting for covariate(s). In univariate analyses, factors robustly associated with severe COVID-19 were male sex (10 studies; pooled RR = 1.73, [95% CI 1.50–2.01]), hypertension (8 studies; 2.87 [2.09–3.93]), diabetes (9 studies; 3.20 [2.26–4.53]), and CVD (10 studies; 4.97 [3.76–6.58]). RR for male sex was likely to be independent of age. For the other three factors, meta-regression analyses suggested confounding by age. Only four studies reported multivariable analysis, but most of them showed adjusted RR ~2 for hypertension, diabetes, and CVD. No study explored renin-angiotensin system inhibitors as a risk factor for severe COVID-19.

**Conclusions:**

Despite the potential for confounding, these results suggest that hypertension, diabetes, and CVD are independently associated with severe COVID-19 and, together with age and male sex, can be informative for predicting the risk of severe COVID-19.

## Introduction

Cases of coronavirus disease 2019 (COVID-19) are rapidly increasing globally. As of April 25, 2020, more than 2.8 million cases have been confirmed and ~200,000 deaths have been reported in ~190 countries [1]. Several studies have rapidly provided crucial data (e.g., incubation period) related to various aspects of the novel coronavirus (SARS-CoV-2: severe acute respiratory syndrome coronavirus 2) infection [2]. However, risk factors for the severity and prognosis of COVID-19 are poorly understood. Such information is critical to identify high risk patients and to facilitate planning (e.g., forecasting the need for hospital beds and mechanical ventilators). These risk factors also have implications for workforce allocation (e.g., assignment of healthcare providers with specific risk factors to positions with reduced risk of exposure to COVID-19).

To date, several studies have reported that a history of cardiovascular disease (CVD) and traditional CVD risk factors, e.g., age, male sex, current smoking, hypertension, and diabetes, are associated with severe COVID-19. However, other than age, results have been inconsistent. Furthermore, few studies that evaluated other potential risk factors accounted for potential confounding by age and sex. For example, some studies reported that hypertension is a risk factor of severe COVID-19, but their observations may simply reflect the fact that hypertension is more common in older adults [3]. Nonetheless, despite the lack of robust evidence, this observation, together with the fact SARS-CoV-2 uses angiotensin-converting enzyme 2 as an entry to human cell [4], has raised a concern about continued use of renin-angiotensin system inhibitors among some clinicians and researchers [5,6].

In this context, we conducted a systematic review of studies reporting cardiovascular risk factors and their relation to severe manifestation of COVID-19 (i.e., death, acute respiratory distress syndrome [ARDS], the need of mechanical ventilator support, and admission to an intensive care unit [ICU]), with a particular interest on studies that adjusted for key confounders such as age and sex.

## Methods

### Search strategy

We conducted this systematic review following the PRISMA Statement. According to the pre-determined protocol, we systematically searched PubMed and Embase for eligible reports (search terms are listed in Web Appendix 1). We included full reports or letters with original data written in English. Eligible study designs were cohort study, cross-sectional, case series, case-control study, and clinical trials. We conducted the literature search on April 3, 2020, and restricted to publications after December 1, 2019. We did not search websites of preprints, because some experts raised a concern about the quality of some papers before a rigorous peer-review process [7].

Our review included studies that reported adult patients, aged 18 years or older. There was no restriction with respect to gender, race/ethnicity, and comorbidities. The primary outcome of interest was severe COVID-19 defined by any of the following: all-cause mortality, ICU admission, ARDS, or the need for mechanical ventilation. We included studies reporting at least 10 cases of severe COVID-19. To obtain reliable estimates with enough number of outcomes and considering clinical cascade (e.g., death as the final outcome), when one study reported results for multiple outcomes, we prioritized any composite outcome followed by ICU admission, ARDS, the need of mechanical ventilation, and mortality.

Potential risk factors of interest were pre-existing CVD (including cardiac disease and cerebrovascular disease) and its traditional risk factors recognized in major CVD clinical guidelines: age, sex, smoking, hypertension, dyslipidemia, and diabetes [8]. We found only one study reporting severity of COVID-19 by dyslipidemia (low-density lipoprotein).

### Study selection

One author (N.D.) conducted the literature search, exported eligible publications to EndNote X8 reference manager (Thomson Reuters, Philadelphia, Pennsylvania), and uploaded them to Covidence (Melbourne, Australia), a platform for literature screening. Eight reviewers (N.D., M.K., Y.G., Y.H., Y.M., X.H., M.C., and J.I.) worked in pairs to independently screen titles and abstracts. For research letters without abstracts, reviewers used text content for the initial screening. All conflicts were resolved by one of two lead reviewers (N.D., M.K.). For all publications accepted at this step, the same pairs performed the full detailed text review to evaluate final eligibility. The two lead reviewers resolved any conflicts.

### Data collection and quality assessment

The same eight reviewers collected relevant data elements from each identified publication and recorded in Microsoft Excel (Microsoft Corporation, Redmond, Washington, USA). Overall quality was based on the Newcastle Ottawa Quality Assessment Scale (NOS) [9], which includes eight items about selection, comparability, and outcome (Web Appendix 2). The NOS score for cohorts’ studies ranges from 0 to 9; a score greater than 6 was considered high-quality. For cross-sectional and case-control studies, we applied an adapted form of the NOS [10]. The maximum score was 10 and 9, and 7 and 6 points were used to identify studies with high quality, respectively. The paired reviewers resolved conflicts related to their own data collection and quality assessment.

### Data synthesis and analysis

We summarized relative risk estimates (odds ratios or hazard ratios) of the association between each risk factor and the primary outcome from the relevant studies. We pooled these estimates using random-effects meta-analysis. When studies did not report these measures of association but the prevalence of risk factors of interest by the outcome status (e.g., survivors vs. non-survivors), we calculated crude odds ratios and their 95% CIs. In this process, when there was any cell with a zero count, we added 0.5 to each cell, as appropriate [11]. We also calculated prediction interval, the range showing the estimate from a new future study would fall in, using pooled estimates of relative risk, its standard error, the number of study included in the meta-analysis, and the estimated between-study heterogeneity τ^2^ [12].

Potential confounding by age and sex is relevant to prior CVD, hypertension, and diabetes, since these comorbidities become more prevalent with increased age [3]. Because most studies did not report adjusted risk estimates for these comorbidities, we conducted meta-regression analyses with random-effects for log relative risk by the difference in mean or median age between those with vs. without primary outcome across eligible studies. For example, if studies with larger age difference between those with vs. those without hypertension tend to have higher relative risk of severe COVID-19 by hypertension, there is the possibility of confounding by age. To obtain reliable estimates, we required at least five studies for each meta-regression analysis. We also depicted funnel plots and visually checked the possibility of publication bias. Heterogeneity of study estimates was assessed by *I*^2^ statistic, and *I*^2^ > 75% was considered high heterogeneity [13].

In our review, we found a total of 10 reports likely using the same data source even though they did not analyze identical samples. Since we could not be certain whether these were duplicate or overlapping publications, we had two approaches to conduct the meta-analyses: (1) a restrictive approach in which we excluded reports with high possibility of overlap [e.g., data from the same hospitals during the same period] and (2) an inclusive approach in which we included all publications. For the restrictive approach, we used the largest study with estimates of interest. To be conservative, we present the restrictive meta-analysis as our primary analysis. The results of the inclusive meta-analysis are available in the supplemental material. A p-value <0.05 was considered statistically significant. All analyses were conducted with Stata 14 or 15 (StataCorp, LLC, College Station, Texas, USA).

## Results

### Search results

**Figure 1:**
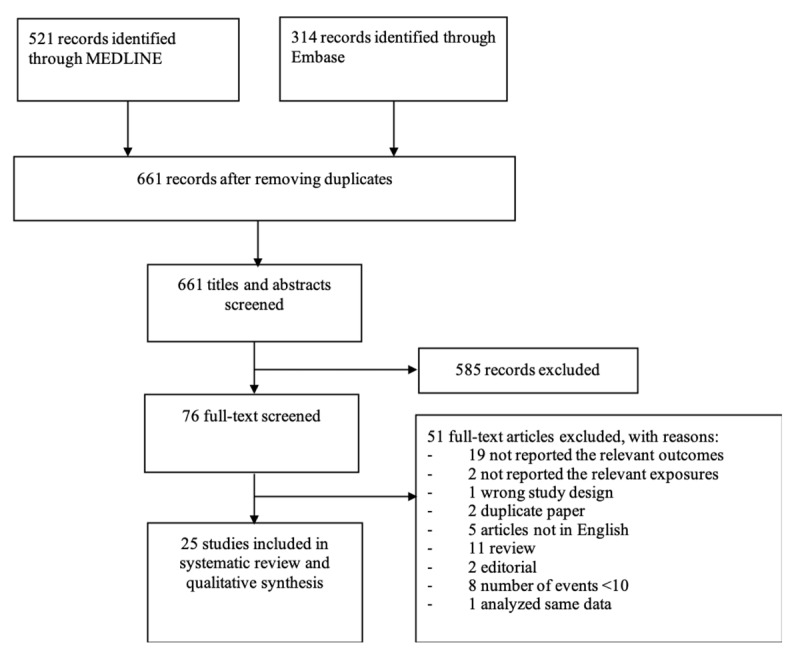
Flow chart of study selection.

Our systematic review identified 661 potentially eligible publications after removing duplicate publications (Figure 1). Of these, 585 publications were excluded after screening titles and abstracts because they were not exploring exposures and outcomes of interest or did not have contrast of interest (e.g., investigating only patients with severe COVID-19). Of the remaining 76 publications reviewed with full-text screening, we excluded 51 publications that did not meet our inclusion criteria, leaving 25 publications for analyses [14,15,16,17,18,19,20,21,22,23,24,25,26,27,28,29,30,31,32,33,34,35,36,37,38]. Most of these publications were considered high quality (Web Table 1). Of the included studies, death was reported in 21 studies, ICU admission in 11 studies, ARDS in 13 studies, and mechanical ventilation in 11 studies. Three studies reported a composite outcome.

### Study characteristics

Most studies reported COVID-19 patients from China (21 studies) and were small with sample size <300 (15 studies) (Table 1). All studies included confirmed COVID-19 patients with laboratory tests. A total of 76,638 COVID-19 patients were included in these studies, with 11,766 patients manifesting severe disease. Most studies exclusively investigated hospitalized COVID-19 cases. Average or median age ranged from 46 to 69 years old, and 45% to 73% of participants were male. Most studies reported the prevalence of CVD risk factors; the prevalence of hypertension ranged from 13% to 41%, diabetes from 5% to 58%, and pre-existing CVD from 1% to 22%.

**Table 1:** Summary of 25 studies included in systematic review.

Reference	Journal	Region	Setting	Sample size	Severe, n (%)	Age median (IQR) or mean (SD) in y	Male, n (%)	Current smoking, n (%)	HTN, n (%)	DM, n (%)	CVD, n (%)

Bhatraju PK	NEJM	USA	9 Seattle-area hospitals	24	18 (75)	64 (18)	15 (63)	5 (22)*	–	14 (58)	2 (8)
Cao J	Intensive Care Med	China	Zhongnan Hospital	102	18 (18)	54 (37–67)	53 (52)	–	28 (28)	11 (11)	5 (5)
Chen J	J Infect	China	Shanghai Public Health Clinical Center	249	22 (9)	51 (36–64)	126 (51)	–	–	–	55 (22)
Chen T	BMJ	China	Tongji Hospital	274	196 (72)	62 (44–70)	170 (62)	11 (4)	93 (34)	47 (17)	22 (8)
Cheng Y	Kidney International	China	Tongji Hospital	701	113 (16)	63 (50–71)	367 (52)	–	233 (33)	100 (14)	
China CDC	CCDC	China	China National Data	44,672	8,255 (19)	–	22,981 (51)	–	2,683 (13)	1,102 (5)	873 (4)
Deng Y	Chin Med J	China	Tongji Hospital and Central Hospital of Wuhan	225	109 (48)	–	124 (55)	–	58 (26)	26 (12)	17 (8)
Guan W	NEJM	China	552 hospitals in 30 provinces	1099	67 (6)	47 (35–58)	637 (58)	137 (13)	165 (15)	81 (7)	27 (3)
Guo T	JAMA Cardiol	China	Seventh Hospital of Wuhan	187	43 (23)	59 (15)	91 (49)	18 (10)	61 (33)	28 (15)	21 (11)
Huang C	Lancet	China	Jinyintan Hospital	41	13 (32)	49 (41–58)	30 (73)	3 (7)	–	–	6 (15)
Lian J	Clin Infect Dis	China	Hospitals in Zhejiang province	788	77 (10)	46 (15)	407 (52)	54 (7)	126 (16)	57 (7)	11 (1)
Liang W	Lancet Oncology	China	575 hospitals across China	1,590	131 (8)	49 ± 16	909 (57)	111 (7)	–	–	–
Onder G	JAMA	Italy	Italy National Data	22,512	1,625 (7)	–	–	–	–	–	–
Ruan Q	Intensive Care Med	China	Jinyintan Hospital and Tongji Hospital	150	67 (45)	–	102 (68)	–	52 (35)	–	13 (9)
Shi S	JAMA Cardiol	China	Zhejiang Province	416	97 (23)	64 (21–95)	205 (49)	–	127 (31)	60 (14)	44 (11)
Tang N	J Thromb Haemost	China	Tongji Hospital	183	21 (12)	54 ± 16	98 (54)	–	–	–	–
US CDC	MMWR	US	US National Data	2,449	121 (5)	–	–	–	–	–	–
US CDC_2	MMWR	USA	US National Data	6,637	457 (6)	–	–	86 (1)	106 (2)	723 (11)	597 (9)
Wang D	JAMA	China	Zhongnan Hospital	138	36 (26)	56 (42–68)	75 (54)	–	43 (31)	14 (10)	20 (15)
Wang L	J Infect	China	Renmin Hospital of Wuhan University	339	80 (24)	69 (65–76)	166 (49)	–	138 (41)	54 (16)	53 (16)
Wu C	JAMA Intern Med	China	Jinyintan Hospital, Wuhan	201	84 (42)	51 (43–60)	128 (64)	–	39 (19)	22 (11)	8 (4)
Yang X	Lancet Respir	China	Jinyintan Hospital, Wuhan	52	35 (67)	60 (13)	35 (67)	2 (4)	–	9 (17)	5 (10)
Yuan M	PLoS One	China	Central Hospital of Wuhan	27	10 (41)	60 (47–69)	12 (45)	–	5 (19)	6 (22)	3 (11)
Zhang L	Ann Oncol	China	Tongji Sino-French New Town Hospital, Union Red Cross Hospital and Union West Hospital	28	15 (54)	65 (56–70)	17 (61)	–	–	4 (14)	4 (14)
Zhou F	Lancet	China	Jinyintan Hospital and Wuhan Pulmonary Hospital	191	59 (31)	56 (46–67)	119 (62)	11 (6)	58 (30)	36 (19)	15 (8)

All studies were published in 2020. **Abbreviations:** COVID-19: coronavirus disease 2019; CVD: cardiovascular disease; DM: diabetes; HTN: hypertension; NR, not reported. * Current or former smoking.

### Sociodemographic factors: age and sex

Older age was associated with higher risk of severe COVID-19 in all 16 studies with relevant estimates (Table 2). Three studies reported the relative risk by age in both unadjusted and multivariable models and did not observe material changes in the effect size of age after adjustment for comorbidities [16,37].

**Table 2:** Relative risk estimates of severe COVID-19 by age in eligible studies.

Study	No. of events	Age, per-year increment	Across categories	

			Reference	Comparison

Bhatraju PK	18 cases with mechanical ventilation	–	<60	Unadjusted OR: 60–70: 3.50 (0.24–51.90) 70–80: NA >80: 0.33 (0.03–3.93)
Chen J	22 cases with ICU admission	Unadjusted OR: 1.08 (1.04–1.13) Adjusted* OR: 1.06 (1.00–1.12)	–	–
Chen T	113 deaths	–	<60	Unadjusted OR: ≥60: 8.55 (4.75–15.40)
Cheng Y	113 deaths	–	≤65	Unadjusted HR: >65: 2.43 (1.66–3.56)
China CDC	1,023 deaths	–	20–29	Unadjusted HR: 30–39: 1.23 (0.51–2.94) 40–49: 2.29 (1.03–5.15) 50–59: 6.79 (3.17–14.54) 60–69: 19.3 (9.1–40.8) 70–79: 44.6 (21.1–94.6) ≥80: 89.4 (42.0–190.5)
Lian J	58 cases with ARDS	–	<60	Unadjusted OR: ≥60: 3.59 (2.04, 6.30)
Liang W	131 composite end points (ICU admission requiring invasive ventilation or death)	Adjusted OR†: 1.048 (1.033–1.064)	–	–
Onder G	1,625 deaths	–	30–39	Unadjusted OR: 40–49: 1.33 (0.42–4.26) 50–59: 3.36 (1.20–9.37) 60–69: 12.05 (4.45–32.63) 70–79: 48.77 (18.20–130.63) ≥80: 84.10 (31.43–225.06)
Shi S	57 deaths	Adjusted HR‡: 1.02 (0.99–1.05)	–	–
US CDC	121 cases with ICU admission	–	20–44	Unadjusted OR: 45–54: 2.80 (1.43–5.49) 55–64: 2.41 (1.21–4.83) 65–74: 4.33 (2.29–8.20) 75–84: 5.78 (2.90–11.51) ≥85: 3.29 (1.40–7.76)
US CDC_2	457 cases with ICU admission	–	19–64	Unadjusted OR: ≥65: 1.21 (0.96–1.52)
Wang L	65 deaths	Unadjusted HR: 1.08 (1.06–1.11)Adjusted HR§: 1.06 (1.03–1.10)	–	–
Wu C	84 cases with ARDS	–	<65	Unadjusted HR: ≥65: 3.26 (2.08–5.11)
Yang X	32 deaths	–	30–49	Unadjusted OR: 50–59: 6.75 (1.16–39.20) 60–69: 5.50 (1.06–28.42) ≥70: 27.00 (2.34–311.17)
Zhang L	15 composite events of ICU admission, use of mechanical ventilation or death	Adjusted HR||: 1.46 (0.48–4.43)	–	–
Zhou F	54 deaths	Unadjusted OR: 1·14 (1·09–1·18) Adjusted OR#: 1.10 (1.03–1.17)	–	–

**Abbreviations:** ARDS: acute respiratory distress syndrome; COVID-19: coronavirus disease 2019; ICU: intensive care unit. * Adjusted for gender, comorbidity, WBC, lymphocytes, CRP, albumin, aspartate aminotransferase, lactate dehydrogenase, eGFR, CD4 cell counts. † Adjusted for age, sex, cancer, HTN, COPD, DM. ‡ Adjusted for age, cardiovascular disease, cerebrovascular disease, diabetes, COPD, renal failure, cancer, ARDS, cardiac injury, creatinine ≥1.50 mg/dL, NT-proBNP ≥900pg/mL. § Adjusted variables unclear. || Adjusted for age, receiving anti-tumor treatment within 14 days, and patchy consolidation on admission CT scan. # Adjusted for age, lymphocyte count, d-dimer, SOFA score, coronary heart disease.

**Figure 2:**
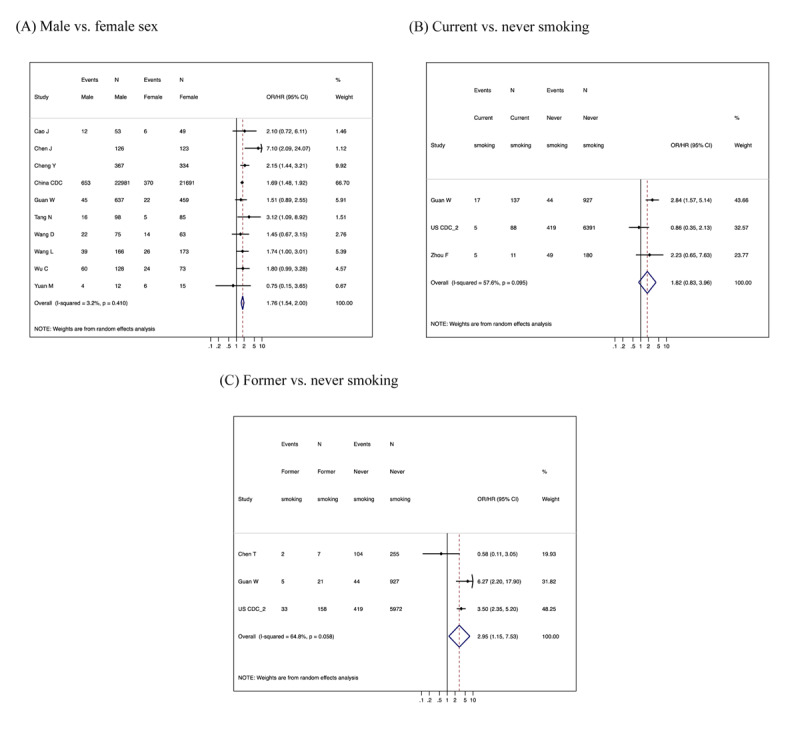
Forest plots of unadjusted relative risk estimates of severe COVID-19 according to gender **(A)**, smoking status **(B)** and **(C)** based on restrictive meta-analysis.

Most studies showed a higher risk of severe COVID-19 in men than in women, with a pooled crude relative risk estimate of severe COVID-19 between men and women of 1.76 (95% CI 1.54–2.00 and prediction interval 1.49–2.08) (Figure 2A and Web Figure 1A). In meta-regression analyses, there was no substantial evidence of confounding by age (Web Figure 2). A funnel plot did not indicate major publication bias (Web Figure 3A).

### Smoking status

Only a couple of studies reported associations of current smoking with severe COVID-19, with only one study reaching statistical significance (Figure 2B and Web Figure 1B). The pooled estimate of relative risk for severe COVID-19 was ~1.8 in both restrictive and inclusive meta-analyses, although statistical significance was restricted to the latter. Three studies reported the association between former versus never smoking, with the pooled relative risk estimates of 2.95 (95% CI 1.15–7.53 and prediction interval 0.15–58.40) (Figure 2C). The corresponding funnel plot is shown in Web Figure 3B.

### Clinical factors: hypertension, diabetes, and prior CVD

**Figure 3:**
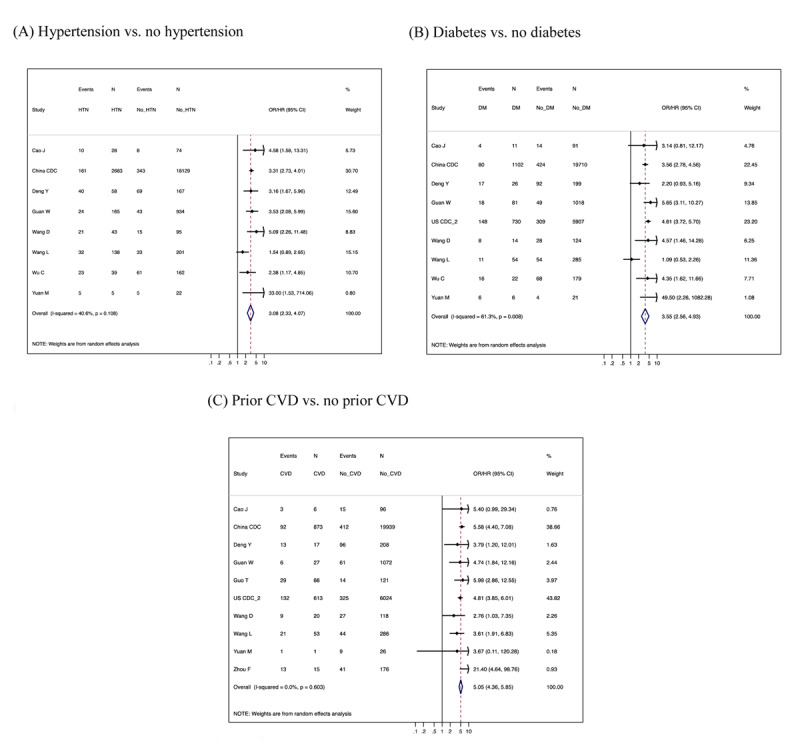
Forest plots of unadjusted relative risk estimates of severe COVID-19 according to hypertension **(A)**, diabetes **(B)**, and prior CVD **(C)** on restrictive meta-analysis.

All eligible studies with data on hypertension reported a positive association of hypertension with severe COVID-19 (Figure 3A and Web Figure 4A), with the primary pooled relative risk estimate of 3.08 (95% CI 2.33–4.07 and prediction interval 1.65–5.75). Most eligible studies also demonstrated a positive association between diabetes and severe COVID-19 (Figure 3B and Web Figure 4B), with the primary pooled relative risk estimate of 3.55 (95% CI 2.56–4.93 and prediction interval 1.56–8.12). Similarly, a majority of eligible studies showed a positive association between prior CVD and severe COVID-19, with the primary pooled relative risk of 5.05 (95% CI 4.36–5.85 and prediction interval 4.36–5.85) (Figure 3C and Web Figure 4C). None of the *I*^2^ in these meta-analyses exceeded 75%, which is a common threshold of high heterogeneity. There was no indication of publication bias for these comorbidities (Web Figure 3C-E). In the one available study that assessed low-density lipoprotein as a risk factor of COVID-19, Wu et al. (2020) reported that higher levels were associated with lower risk of severe COVID-19 (0.63 [0.44–0.88] per 1 mmol/L increment) [34]; no other lipid fractions were reported. No study explored renin-angiotensin system inhibitors as a risk factor for severe COVID-19.

### Potential confounding by age and sex

**Figure 4:**
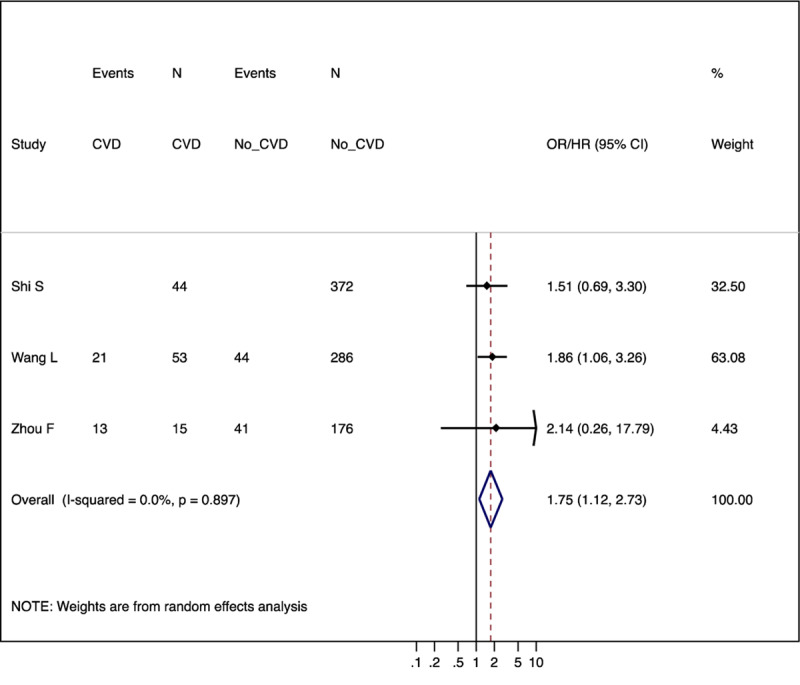
Forest plots of adjusted relative risk estimates of severe COVID-19 according to prior CVD.

Among the identified studies, only four studies reported adjusted relative risk estimates of these clinical factors. For pre-existing CVD, the pooled adjusted relative risk estimate across three studies was 1.75 (95% CI 1.12–2.73 and prediction interval 1.12–2.73) (Figure 4). Although these studies accounted for different confounders, all included age. Two studies reported adjusted relative risk for diabetes, but one of them was likely to have an over adjustment bias by including potential mediators such as kidney failure, ARDS, and end organ damage markers (natriuretic peptide, cardiac troponin, and serum creatinine). The other study by Liang et al. (2020) with 1,590 patients reported an adjusted odds ratio of 2.21 (1.33–3.66) for diabetes [25]. This study by Liang et al. (2020) also showed the adjusted odds ratio of 1.88 (1.22–2.90) for hypertension.

**Figure 5:**
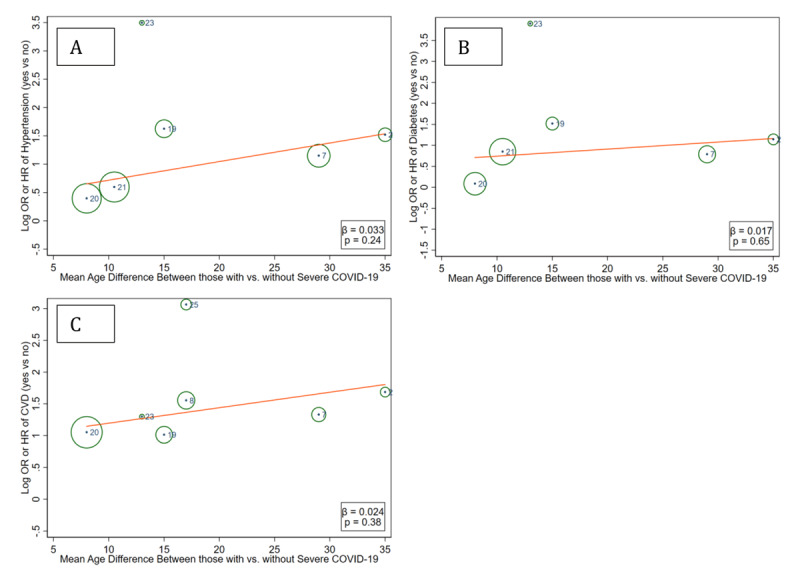
Meta-regression of unadjusted relative risk of severe COVID-19 for hypertension (A), diabetes (B), and CVD (C) by age difference between severe vs. non-severe COVID-19 based on restrictive meta-analysis.

Meta-regression analyses demonstrated that studies with greater age difference between those with versus without severe COVID-19 tended to have greater relative risk according to the presence of hypertension, diabetes, and pre-existing CVD, indicating some levels of potential confounding by age (Figure 5 and Web Figure 5), although the statistical significance was only seen for hypertension in the inclusive meta-analysis (Web Figure 5A). The meta-regression analysis did not indicate that a higher proportion of male sex confounded the association of these clinical factors with severe COVID-19 (Web Figure 6).

## Discussion

To our knowledge, this is the first systematic review and meta-analysis focusing on the relationship of severe COVID-19 with CVD and its risk factors. We confirmed a robust association of age and male sex with severe COVID-19. Their contributions are likely to be independent of each other. A few studies demonstrated positive associations of current and former smoking with severe COVID-19. Several studies reported that pre-existing CVD, hypertension, and diabetes were also associated with severe COVID-19. However, only four studies reported estimates for these comorbidities adjusted for age and/or sex. Across those studies, we could only meaningfully pool estimates for pre-existing CVD, which demonstrated a significant relative risk of ~1.8. One relatively large study by Liang et al. (2020) showed independent associations of hypertension and diabetes with severe COVID-19 in analyses that adjusted for age and a few other comorbidities [25]. Although the primary estimate was not statistically significant, our meta-regression analyses indicated some degree of confounding by age, but not necessarily by sex, for the associations of hypertension, diabetes, and prior CVD with severe COVID-19.

Regarding age, the pattern was consistent in national surveys database in the US, China, and Italy, without any clear thresholds (Web Table 2). The case fatality rate in those three databases exceeded 1% around age of 50–55 years and 10% above 80–85 years (above 70 years in Italy). The positive association of sociodemographic factors (age and male sex) with severe COVID-19 is consistent with reports of other infectious diseases (e.g., influenza virus and SARS in 2003) [39,40,41]. Older age is linked to reduced immune reaction, more comorbidities, and limited organ reserve [3,39,42]. Male sex is related to higher prevalence of comorbidities, less frequency of washing hands, and immunological disadvantage given X-chromosome coding proteins in the immune system [2,43,44], whereas smoking can damage respiratory system [45].

In our meta-analysis, we confirmed overall positive crude associations of CVD, hypertension, and diabetes with severe COVID-19, with pooled relative risk estimates around 3-5. An important question is whether these associations are independent of major confounders, particularly age. In this regard, the meta-analysis of three studies indicated an independent association for pre-existing CVD. For hypertension and diabetes, we could not obtain a definite answer as very few studies ran multivariable models, although our meta-regression analysis indicated some degree of confounding by age. Of note, Liang et al, (2020) showed independent associations of hypertension and diabetes with severe COVID-19 in a relatively large sample size of 1,590 patients. There is also biologic plausibility. Hypertension and diabetes are leading risk factors for CVD and kidney diseases, and there is evidence that COVID-19 damages these organs [28,46].

However, we should not conflate the observed associations of hypertension and diabetes with severity of COVID-19 infection as indicative of adverse effects of renin-angiotensin system inhibitors. None of the 25 studies in our systematic review explored these medications regarding severe COVID-19. Moreover, in the only study reporting the prevalence of renin-angiotensin system inhibitors use [18], ~30% of patients reported prevalent hypertension, but only ~5% of patients were taking renin-angiotensin system inhibitors [18]. Thus, it is reasonable that many expert organizations recommend continuing this category of medications until better evidence becomes available [4].

Our results can potentially be used to guide decision-making. While the lack of discrete age thresholds for severe COVID-19 complicates this process, data on case fatality rates from three countries (Web Table 2) suggest that age older than 60 or 65 years confers high risk of severe COVID-19 (relative risk > ~5 compared to <50 years). Our results also indicate male sex is an independent risk factor with a pooled relative risk of ~1.8. Although the pooled crude relative risk of ~3–5 for hypertension, diabetes, and CVD probably overestimate their impact beyond age and sex, all or some of them are likely to each confer 1.5–2 times greater risk. Thus, using these factors, it is possible to estimate, at least crudely, the risk of severe COVID-19. For example, a man aged 60-65 years old, with either hypertension, diabetes, or prior CVD would have a risk of ~15 fold higher risk (approximation of 5 × 1.8 × 2) compared to a woman younger than 50 years without any of these clinical factors. Such information could be used to inform decisions on testing for COVID-19, clinical management of COVID-19, and workforce planning.

Our study has some limitations. First, reflecting the fact that the outbreak started from China, most studies were from China. However, given similar case-fatality rates and clinical manifestations across different countries, it seems likely that these results are largely generalizable. Nonetheless, we need to acknowledge regional variations of some risk factors (e.g., ~25-fold difference in the prevalence of smoking in men vs. women in China) [47] and thus future investigations in different regions would be valuable. Second, we did not include non-English publications and missed some relevant papers particularly written in Chinese especially in the beginning of outbreak. Third, most studies reported odds ratios, which are known to overestimate risk ratio when the prevalence of exposures is relatively high. Fourth, we cannot deny the possibility that some patients were included in multiple studies especially in the China CDC report and other Chinese studies [19]. Nonetheless, the pooled estimates were largely similar in analyses that excluded the China CDC data (data not shown). Finally, the literature of COVID-19 is growing rapidly, and thus there is a lag time from our literature search and publication.

On the other hand, our systematic review has several strengths: in-depth review of CVD and its risk factors, a clinically relevant definitions of severe COVID-19 that minimize subjective reporting, careful consideration of potential overlap of patients by conducting restrictive and inclusive meta-analyses, meta-regression to explore potential confounding, and relatively short elapsed time of ~3 weeks between the literature search and manuscript submission.

In conclusion, our systematic review and meta-analysis found associations of older age and male sex as risk factors of severe COVID-19. Furthermore, despite the potential for confounding, our review suggests that hypertension, diabetes, and CVD are associated with severe COVID-19. These demographic and clinical factors can be informative for predicting the risk of severe COVID-19.

## Additional File

The additional file for this article can be found as follows:

10.5334/gh.814.s1 Appendix. Web tables and figures.
