# Supplementary material for: The Relationship of COVID-19 Severity with Cardiovascular Disease and Its Traditional Risk Factors: A Systematic Review and Meta-Analysis
Source: Glob Heart. 2020 Sep 22;15(1):64. doi: 10.5334/gh.814 (PMC7546112; doi:10.5334/gh.814)
Supplement: Appendix. — Web tables and figures. [file gh-15-1-814-s1.pdf]

Web Table 1. Quality assessment of the 25 included studies with the Newcastle-Ottawa Scale

**Cohort study**						
**Reference**	**Year**	**Journal**	**Selection/4**	**Comparability/2**	**Outcome/3**	**Total score/9**
Bhatraju PK	2020	N Engl J Med	3	0	3	6
Cao J	2020	Intensive Care Med	3	0	3	6
Chen J	2020	J Infect	3	2	1	6
Cheng Y	2020	Kidney International	4	2	3	9
Deng Y	2020	Chin Med J (Engl)	3	0	3	6
Huang C	2020	Lancet	4	0	3	7
Lian J	2020	Clin Infect Dis	4	0	3	7
Liang W	2020	The Lancet Oncology	4	1	3	8
Ruan Q	2020	Intensive Care Med	3	0	2	5
Shi S	2020	JAMA Cardiol	3	2	2	7
Tang N	2020	J Thromb Haemost	4	0	3	7
Wang D	2020	JAMA	4	0	3	7
Wang L	2020	J Infect Mar	4	1	3	8
Wu C	2020	JAMA Intern Med	3	0	2	5
Yang X	2020	Lancet Respir	3	0	2	5
Yuan M	2020	PLoS One	3	0	2	5
Zhang L	2020	Ann Oncol	3	1	3	7
Zhou F	2020	Lancet	3	1	2	6
**Cross-sectional study**						
**Reference**	**Year**	**Journal**	**Selection/5**	**Comparability/2**	**Outcome/3**	**Total score/10**
China CDC	2020	CCDC Weekly	5	0	3	8
Onder G	2020	JAMA	5	0	2	7
US CDC_1	2020	MMWR	5	0	3	8
US CDC_2	2020	MMWR Morb Mortal Wkly Rep	3	0	1	4

**Case Control study***						
**Reference**	**Year**	**Journal**	**Selection/4**	**Comparability/2**	**Exposure/3**	**Total score/9**
Chen T	2020	BMJ	3	0	3	6
Guan W	2020	Eur Respir J	3	0	2	5
Guo T	2020	JAMA Cardiol	3	0	2	5

*These studies included patients discharged dead and those discharged alive (patients still at hospitals were excluded). Since these studies identified patients based on the outcome status and explored exposures retrospectively, we categorized them as case-control study.

Web Table 2. Case fatality ratios by age categories in national databases from US, China, and Italy

US CDC				China CDC				Italy NIH			
Age category	# of cases	# of deaths*	CFR	Age category	# of cases	# of deaths	CFR	Age category	# of cases*	# of deaths	CFR
0-19	123	0	0	0-9	416	0	0	0-9		0	0
				10-19	549	1	0.2	10-19	†	0	0
20-44	705	1	0.1	20-29	3619	7	0.2	20-29		0	0
				30-39	7600	18	0.2	30-39	1333	4	0.3
45-54	429	2	0.5	40-49	8571	38	0.4	40-49	2500	10	0.4
55-64	429	6	1.4	50-59	10008	130	1.3	50-59	4300	43	1.0
65-74	409	11	2.7	60-69	8583	309	3.6	60-69	3971	139	3.5
75-84	210	9	4.3	70-79	3918	312	8	70-79	4516	578	13
≥85	144	15	10	≥80	1408	208	15	≥80	4208	850	20
total	2449	44	1.8	total	44672	1023	2.3	total	†	1625	7.2

*Not reported but calculated by the authors of the current systematic review

†Unable to calculate.

Abbreviations: CDC, Centers for Disease Control and Prevention; CFR, case fatality ratios; NIH, National Institutes of Health.

Web Figure 1. Forest plots of relative risk estimates of severe COVID-19 according to male vs. female sex (A) and current vs. non-current smoking (B) based on inclusive meta-analysis

(A) Male vs. female sex

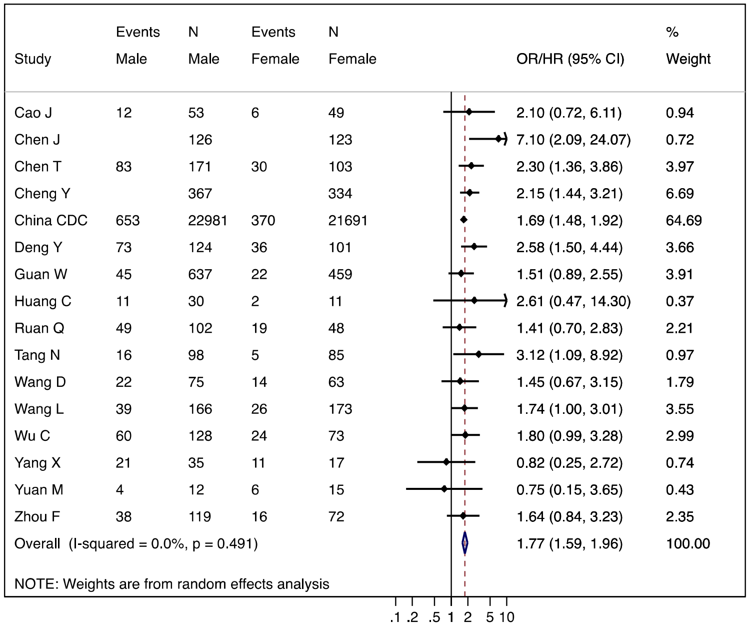

(B) Current vs. never smoking

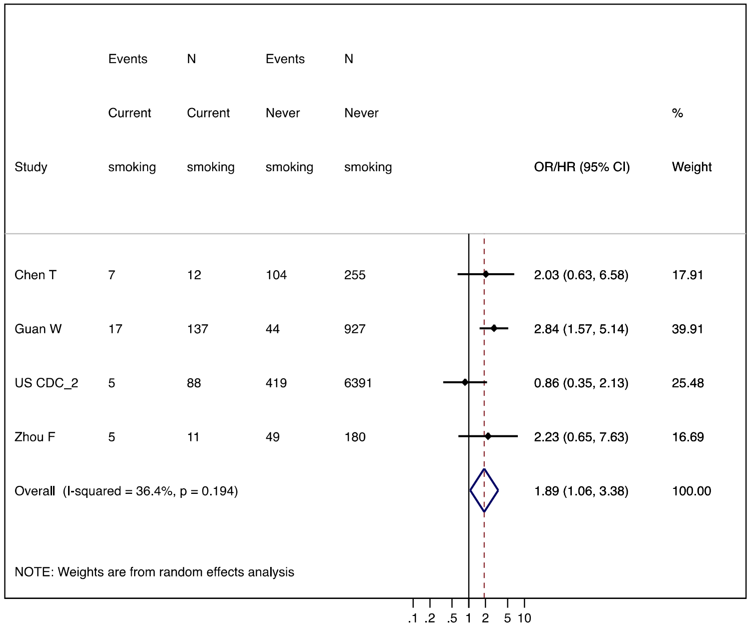

Prediction interval for figure 1A (1.59-1.96) and 1B (0.53-6.77).

Blank space for Events and N indicates that the original study did not report relevant information.

Web Figure 2. Meta-regression of relative risk of severe COVID-19 for male vs. female gender by age difference between severe vs. non-severe COVID-19

(A) Restrictive

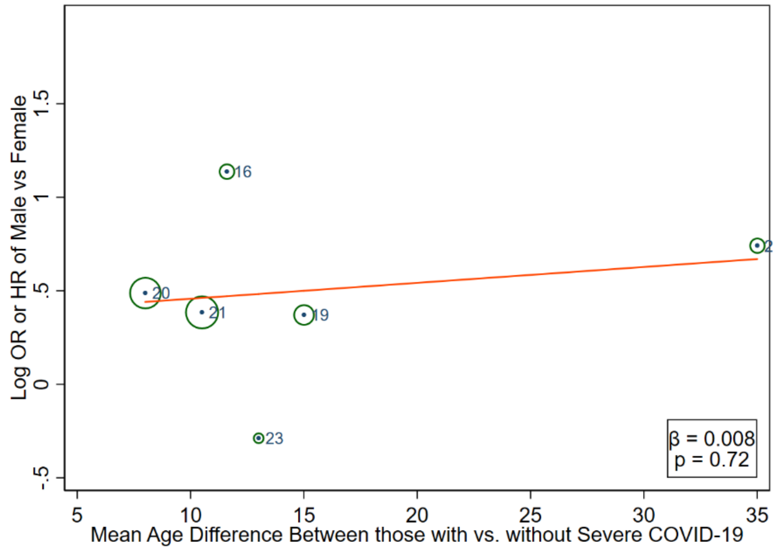

(B) Inclusive

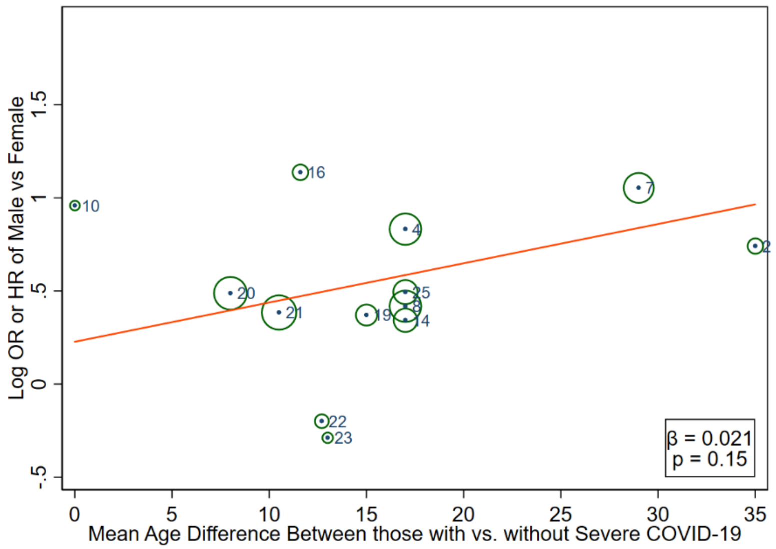

List of studies: 1 Bhatraju PK et al, N Engl J Med; 2 Cao J et al, Intensive Care Med; 3 Chen J et al, J Infect; 4 Chen T et al, BMJ; 5 Cheng Y et al, Kidney International; 6 China CDC, CCDC Weekly; 7 Deng Y et al, Chin Med J (Engl); 8 Guan W et al, N Eng J Med; 9 Guo T et al, JAMA Cardiol; 10 Huang C et al, Lancet; 11 Lian J et al, The Lancet Oncology; 12 Liang W et al, The Lancet Oncology; 13 Onder G et al, JAMA; 14 Ruan Q et al, Intensive Care Med; 15 Shi S et al, JAMA Cardiol; 16 Tang N et al, J Thromb Haemost; 17 US CDC, MMWR; 18 US CDC_2, MMWR; 19 Wang D et al, JAMA; 20 Wang L et al, J Infect Mar; 21 Wu C et al, JAMA Intern Med; 22 Yang X et al, Lancet Respir; 23 Yuan M et al, PLoS One; 24 Zhang L et al, Ann Oncol; 25 Zhou F et al, Lancet

Web Figure 3. Funnel plots for relative risk estimates of severe COVID-19 for male gender, smoking, hypertension, diabetes, and prior CVD

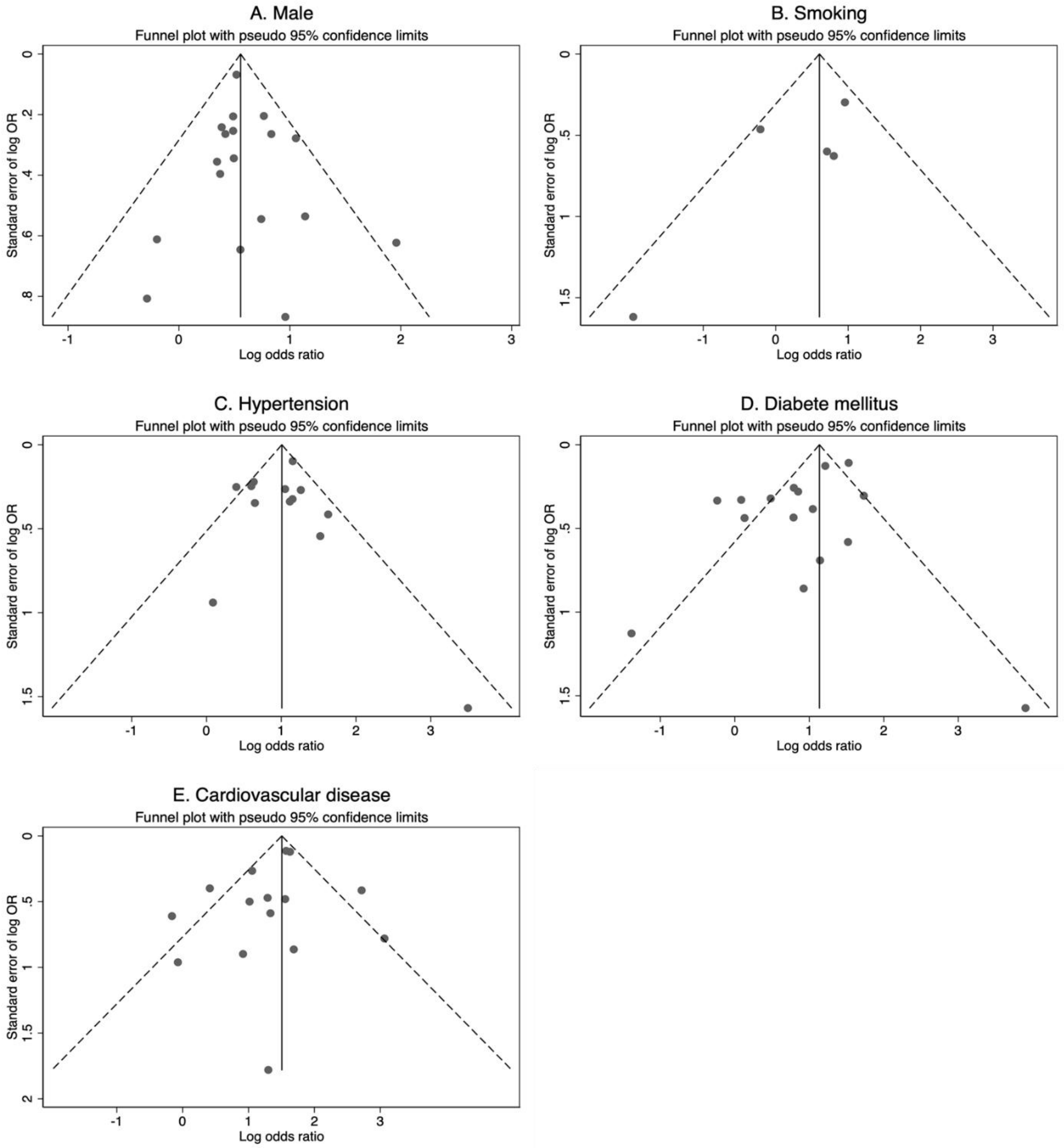

Web Figure 4. Forest plots of unadjusted relative risk estimates of severe COVID-19 according to hypertension (A), diabetes (B), and prior CVD (C) based on inclusive meta-analysis

(A) Hypertension vs. no hypertension

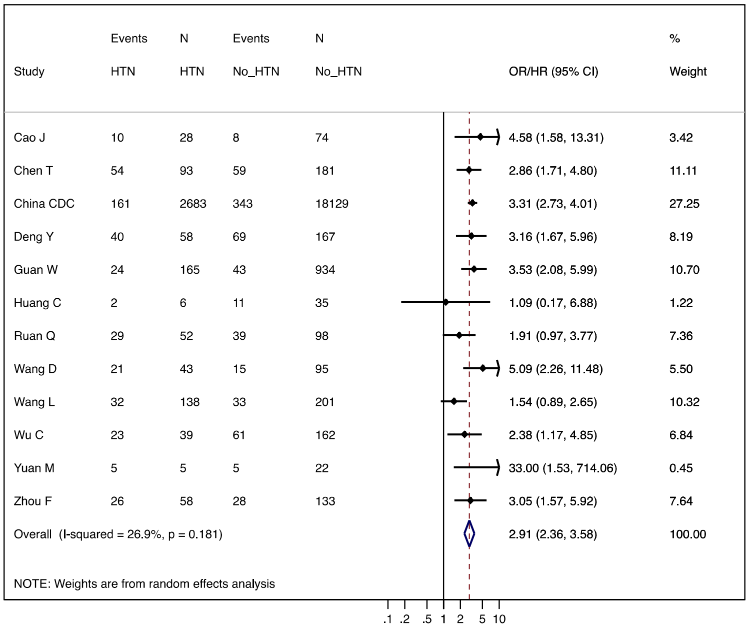

(B) Diabetes vs. no diabetes

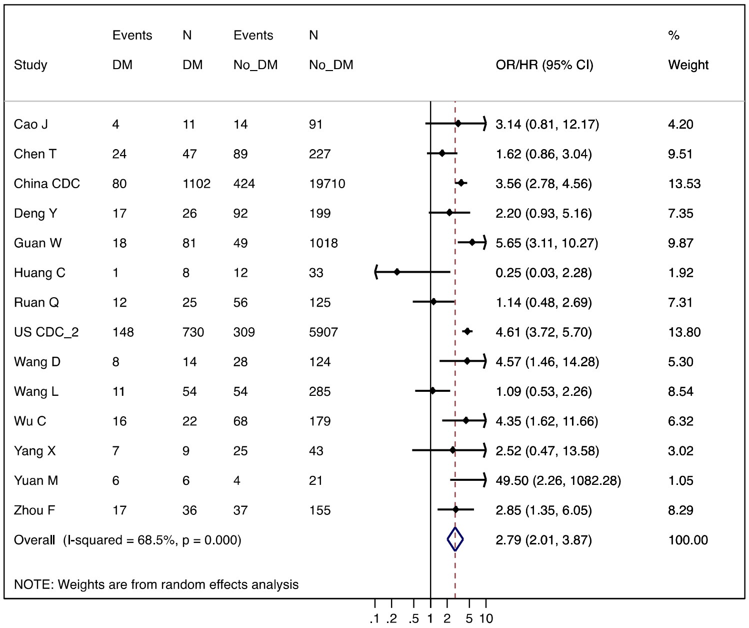

(C) Prior CVD vs. no prior CVD

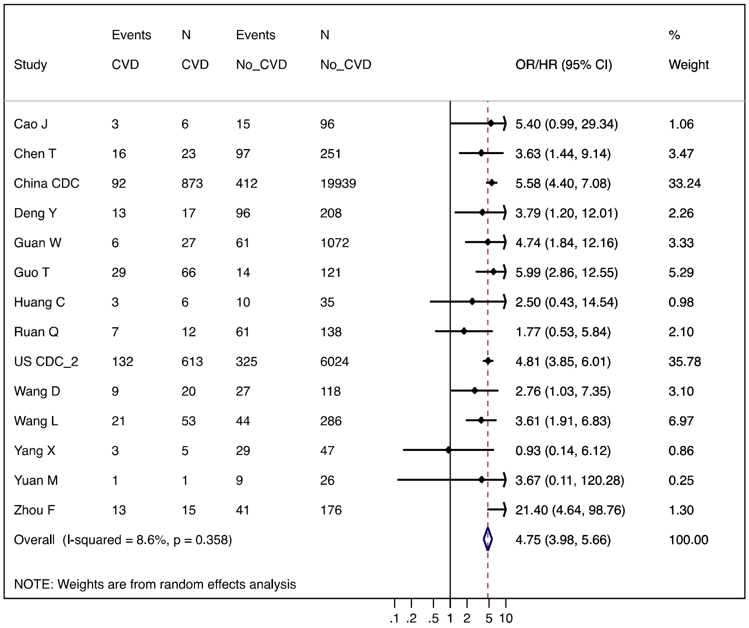

Prediction intervals for figure 4A (1.87-4.52), 4B (1.02-7.58), and 4C (3.61-6.24).

Blank space for Events and N indicates that the original study did not report relevant information.

Web Figure 5. Meta-regression of unadjusted relative risk of severe COVID-19 for hypertension (A), diabetes (B), and CVD (C) by age difference between severe vs. non-severe COVID-19 based on inclusive meta-analysis

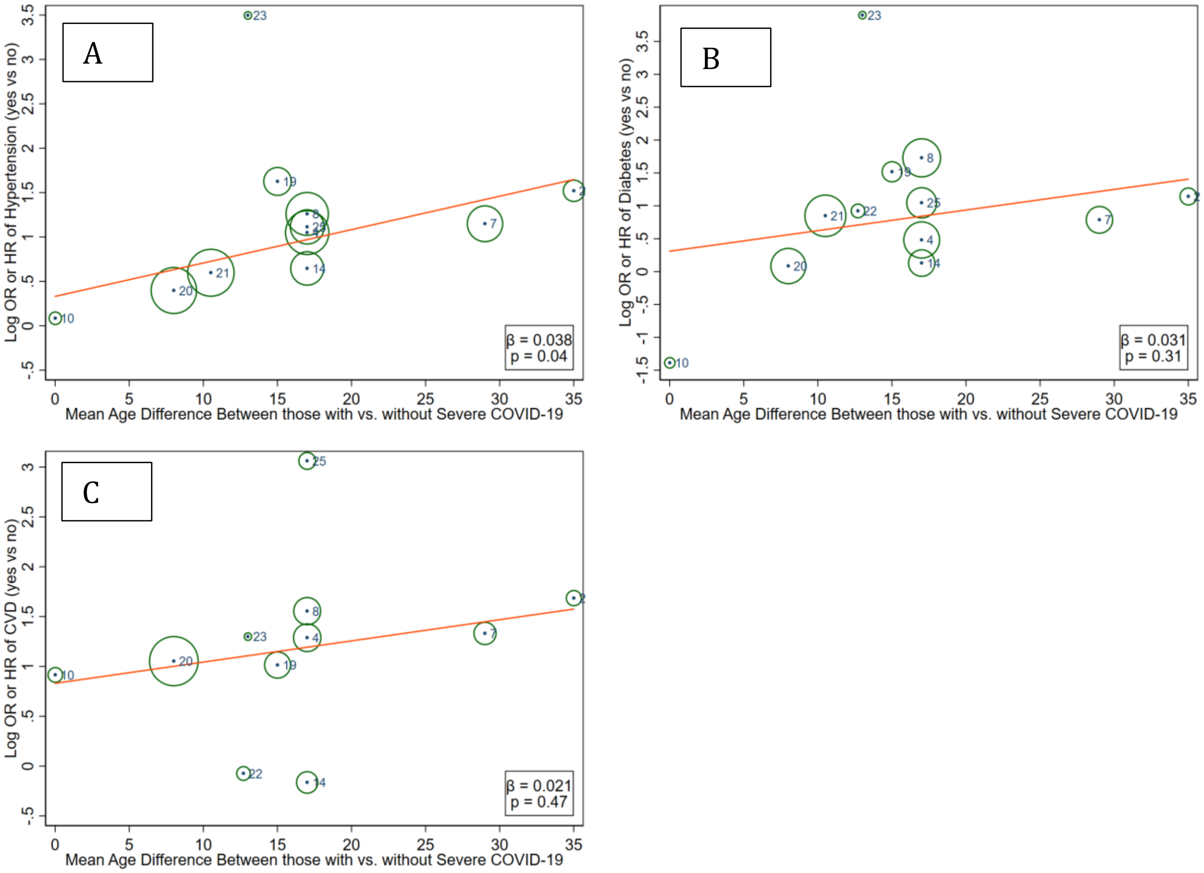

List of studies: 1 Bhatraju PK et al, N Engl J Med; 2 Cao J et al, Intensive Care Med; 3 Chen J et al, J Infect; 4 Chen T et al, BMJ; 5 Cheng Y et al, Kidney International; 6 China CDC, CCDC Weekly; 7 Deng Y et al, Chin Med J (Engl); 8 Guan W et al, N Eng J Med; 9 Guo T et al, JAMA Cardiol; 10 Huang C et al, Lancet; 11 Lian J et al, The Lancet Oncology; 12 Liang W et al, The Lancet Oncology; 13 Onder G et al, JAMA; 14 Ruan Q et al, Intensive Care Med; 15 Shi S et al, JAMA Cardiol; 16 Tang N et al, J Thromb Haemost; 17 US CDC, MMWR; 18 US CDC_2, MMWR; 19 Wang D et al, JAMA; 20 Wang L et al, J Infect Mar; 21 Wu C et al, JAMA Intern Med; 22 Yang X et al, Lancet Respir; 23 Yuan M et al, PLoS One; 24 Zhang L et al, Ann Oncol; 25 Zhou F et al, Lancet

Web Figure 6. Meta-regression of relative risk estimates of severe COVID-19 for hypertension, diabetes, and CVD by the difference in the proportion of male sex between those with vs. those without severe COVID-19

Restrictive analysis

Hypertension

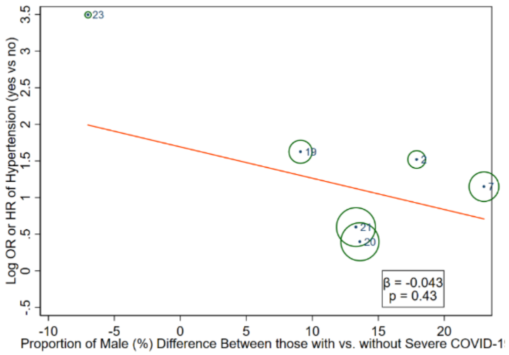

Diabetes

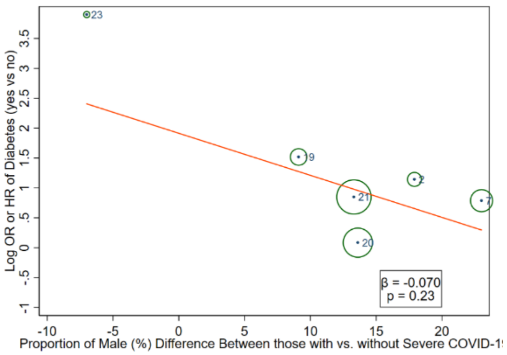

Pre-existing CVD

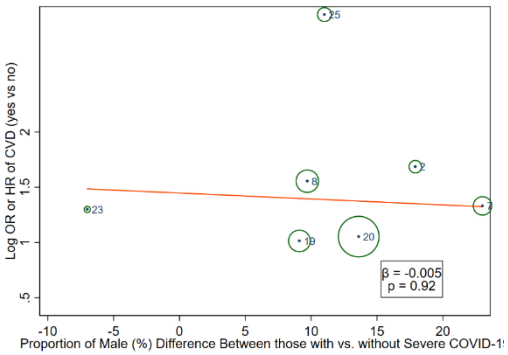

Inclusive analysis

Hypertension

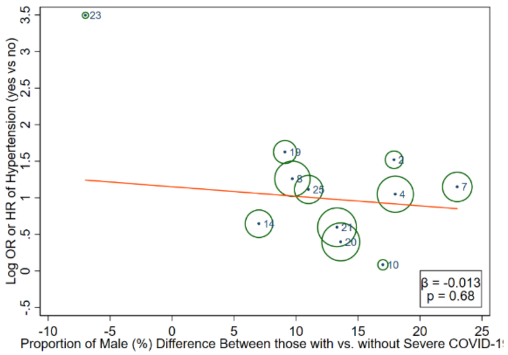

Diabetes

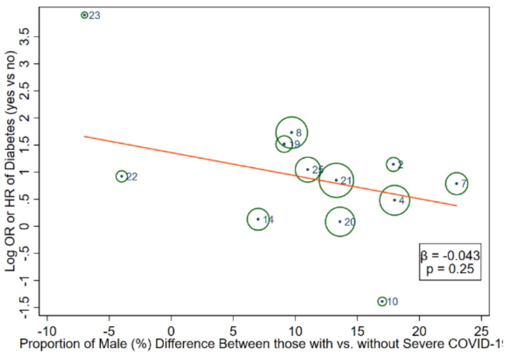

Pre-existing CVD

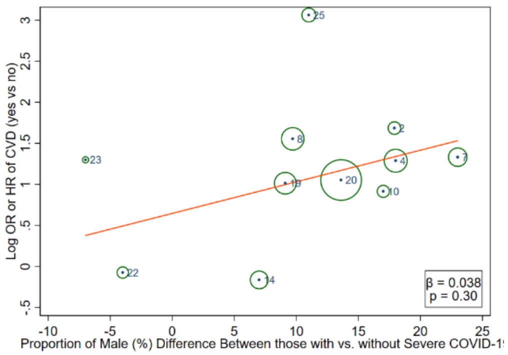

List of studies: 1 Bhatraju PK et al, N Engl J Med; 2 Cao J et al, Intensive Care Med; 3 Chen J et al, J Infect; 4 Chen T et al, BMJ; 5 Cheng Y et al, Kidney International; 6 China CDC, CCDC Weekly; 7 Deng Y et al, Chin Med J (Engl); 8 Guan W et al, N Eng J Med; 9 Guo T et al, JAMA Cardiol; 10 Huang C et al, Lancet; 11 Lian J et al, The Lancet Oncology; 12 Liang W et al, The Lancet Oncology; 13 Onder G et al, JAMA; 14 Ruan Q et al, Intensive Care Med; 15 Shi S et al, JAMA Cardiol; 16 Tang N et al, J Thromb Haemost; 17 US CDC, MMWR; 18 US CDC_2, MMWR; 19 Wang D et al, JAMA; 20 Wang L et al, J Infect Mar; 21 Wu C et al, JAMA Intern Med; 22 Yang X et al, Lancet Respir; 23 Yuan M et al, PLoS One; 24 Zhang L et al, Ann Oncol; 25 Zhou F et al, Lancet

Web Appendix 1. Searching strategy

PubMed:

(((("coronavirus"[MeSH Terms] OR "coronavirus"[All Fields]) OR ("severe acute respiratory syndrome coronavirus 2"[Supplementary Concept] OR "severe acute respiratory syndrome coronavirus 2"[All Fields] OR "2019 ncov"[All Fields])) OR ("COVID-19"[All Fields] OR "severe acute respiratory syndrome coronavirus 2"[Supplementary Concept] OR "severe acute respiratory syndrome coronavirus 2"[All Fields] OR "2019-nCoV"[All Fields] OR "SARS-CoV-2"[All Fields] OR "2019nCoV"[All Fields] OR (("Wuhan"[All Fields] AND ("coronavirus"[MeSH Terms] OR "coronavirus"[All Fields])) AND 2019/12[PDAT] : 2030[PDAT]))) OR ("severe acute respiratory syndrome coronavirus 2"[Supplementary Concept] OR "severe acute respiratory syndrome coronavirus 2"[All Fields] OR "sars cov 2"[All Fields])) AND (((((((("death"[MeSH Terms] OR "death"[All Fields]) OR ("mortality"[Subheading] OR "mortality"[All Fields] OR "mortality"[MeSH Terms])) OR ("survivors"[MeSH Terms] OR "survivors"[All Fields] OR "survivor"[All Fields])) OR "icu"[All Fields]) OR ("intensive care units"[MeSH Terms] OR ("intensive"[All Fields] AND "care"[All Fields] AND "units"[All Fields]) OR "intensive care units"[All Fields] OR ("intensive"[All Fields] AND "care"[All Fields] AND "unit"[All Fields]) OR "intensive care unit"[All Fields])) OR ("ventilators, mechanical"[MeSH Terms] OR ("ventilators"[All Fields] AND "mechanical"[All Fields]) OR "mechanical ventilators"[All Fields] OR "ventilator"[All Fields])) OR ARDS[All Fields]) OR ("respiratory distress syndrome, adult"[MeSH Terms] OR ("respiratory"[All Fields] AND "distress"[All Fields] AND "syndrome"[All Fields] AND "adult"[All Fields]) OR "adult respiratory distress syndrome"[All Fields] OR ("acute"[All Fields] AND "respiratory"[All Fields] AND "distress"[All Fields] AND "syndrome"[All Fields]) OR "acute respiratory distress syndrome"[All Fields])) OR ("respiratory insufficiency"[MeSH Terms] OR ("respiratory"[All Fields] AND "insufficiency"[All Fields]) OR "respiratory insufficiency"[All Fields] OR ("respiratory"[All Fields] AND "failure"[All Fields]) OR "respiratory failure"[All Fields])) AND ("2019/12/01"[PDAT] : "2020/04/03"[PDAT])

Embase:

('coronavirus'/exp OR '2019 ncov' OR 'covid 19' OR 'sars cov 2')
AND
('death'/exp OR death OR 'mortality'/exp OR mortality OR 'survivor'/exp OR survivor OR icu OR (intensive AND ('care'/exp OR care) AND ('unit'/exp OR unit)) OR 'ventilator'/exp OR ventilator OR 'ards'/exp OR ards OR (acute AND respiratory AND ('distress'/exp OR distress) AND ('syndrome'/exp OR syndrome)) OR (respiratory AND ('failure'/exp OR failure)))

Web Appendix 2. Newcastle Ottawa Scales

Newcastle Ottawa Scales for Cohort Studies

Note: A study can be awarded a maximum of one star for each numbered item within the Selection and Outcome categories. A maximum of two stars can be given for Comparability.

Selection

1) Representativeness of the exposed cohort

a) truly representative of the average ________________ (describe) in the community *

b) somewhat representative of the average _______________ in the community *

c) selected group of users (e.g. nurses, volunteers)

d) no description of the derivation of the cohort

2) Selection of the non-exposed cohort

a) drawn from the same community as the exposed cohort *

b) drawn from a different source

c) no description of the derivation of the non-exposed cohort

3) Ascertainment of exposure

a) secure record (e.g. surgical records) *

b) structured interview *

c) written self report

d) no description

4) Demonstration that outcome of interest was not present at start of study

a) yes *

b) no

Comparability

1) Comparability of cohorts on the basis of the design or analysis

a) study controls for _______________ (select the most important factor) *

b) study controls for any additional factor * (This criterion could be modified to indicate specific control for a second important factor.)

Outcome

1) Assessment of outcome

a) independent blind assessment *

b) record linkage *

c) self report

d) no description

2) Was follow-up long enough for outcomes to occur

a) yes (select an adequate follow up period for outcome of interest) *

b) no

3) Adequacy of follow up of cohorts

a) complete follow up - all subjects accounted for *

b) subjects lost to follow up unlikely to introduce bias - small number lost - > ____ % (select an adequate %) follow up, or description provided of those lost) *

c) follow up rate < ____% (select an adequate %) and no description of those lost

d) no statement

Newcastle-Ottawa Scale Adapted for Cross-sectional Studies

Selection: (Maximum 5 stars)

1) Representativeness of the sample:

a) Truly representative of the average in the target population. * (all subjects or random sampling)

b) Somewhat representative of the average in the target population. * (non-random sampling)

c) Selected group of users.

d) No description of the sampling strategy.

2) Sample size:

a) Justified and satisfactory. *

b) Not justified.

3) Non-respondents:

a) Comparability between respondents and non-respondents characteristics is established, and the response rate is satisfactory. *

b) The response rate is unsatisfactory, or the comparability between respondents and non-respondents is unsatisfactory.

c) No description of the response rate or the characteristics of the responders and the non-responders.

4) Ascertainment of the exposure (risk factor):

a) Validated measurement tool. **

b) Non-validated measurement tool, but the tool is available or described. *

c) No description of the measurement tool.

Comparability: (Maximum 2 stars)

1) The subjects in different outcome groups are comparable, based on the study design or analysis. Confounding factors are controlled.

a) The study controls for the most important factor (select one). *

b) The study control for any additional factor. *

Outcome: (Maximum 3 stars)

1) Assessment of the outcome:

a) Independent blind assessment. **

b) Record linkage. **

c) Self report. *

d) No description.

2) Statistical test:

a) The statistical test used to analyze the data is clearly described and appropriate, and the measurement of the association is presented, including confidence intervals and the probability level (p value). *

b) The statistical test is not appropriate, not described or incomplete.

Newcastle Ottawa Scales for Case Control Studies

Note: A study can be awarded a maximum of one star for each numbered item within the Selection and Exposure categories. A maximum of two stars can be given for Comparability.

Selection

1) Is the case definition adequate?

a) yes, with independent validation *

b) yes, eg record linkage or based on self reports

c) no description

2) Representativeness of the cases

a) consecutive or obviously representative series of cases *

b) potential for selection biases or not stated

3) Selection of Controls

a) community controls *

b) hospital controls

c) no description

4) Definition of Controls

a) no history of disease (endpoint) *

b) no description of source

Comparability

1) Comparability of cases and controls on the basis of the design or analysis

a) study controls for _______________ (Select the most important factor.) *

b) study controls for any additional factor * (This criteria could be modified to indicate specific

control for a second important factor.)

Exposure

1) Ascertainment of exposure

a) secure record (eg surgical records) *

b) structured interview where blind to case/control status *

c) interview not blinded to case/control status

d) written self report or medical record only

e) no description

2) Same method of ascertainment for cases and controls

a) yes *

b) no

3) Non-Response rate

a) same rate for both groups*

b) non respondents described

c) rate different and no designation
